# Metagenomic next-generation sequencing improves diagnosis of *Talaromyces marneffei* and mixed infections in HIV/AIDS patients: a retrospective study

**DOI:** 10.3389/fmed.2026.1800314

**Published:** 2026-04-07

**Authors:** Xiaodi Li, Dabiao Chen, Yunfei Xiao, Ziying Lei, Xiaohua Yang, Yeqiong Zhang, Lili Li, Yue Zheng, Ying Zhang, Zhanlian Huang, Bingliang Lin

**Affiliations:** 1 Department of Infectious Diseases, Third Affiliated Hospital of Sun Yat-sen University, Guangzhou, Guangdong, China; 2 Guangdong Key Laboratory of Liver Disease Research, Third Affiliated Hospital of Sun Yat-sen University, Guangzhou, Guangdong, China

**Keywords:** diagnosis, HIV/AIDS, metagenomic next-generation sequencing, mixed infection, *Talaromyces marneffei*

## Abstract

**Background:**

Opportunistic infections remain a leading cause of morbidity in people living with HIV (PLWH). *Talaromyces marneffei* (*T. marneffei*) accounts for up to 15% of HIV-related hospitalizations in endemic regions. Metagenomic next-generation sequencing (mNGS) offers rapid pathogen detection; however, its utility in diagnosing HIV-associated coinfections is uncertain.

**Methods:**

This retrospective study enrolled 56 hospitalized PLWH with coinfections at the Third Affiliated Hospital of Sun Yat-sen University from March 2022 to October 2024. All patients underwent pathogen detection using both mNGS and CTM, with their diagnostic performance compared. Clinical data, treatment adjustments, and outcomes were analyzed.

**Results:**

mNGS demonstrated significantly higher detection rate (84.4%, 54/64; 95% CI: 73.1–92.2%) than CTM (28.1%, 18/64; 95% CI: 17.6–40.8%; *p* < 0.0001), especially for *T. marneffei* detection (100% vs. 45.5%, *p* < 0.0001). mNGS identified *T. marneffei* in 39.3% (*n* = 22/56) of patients, including two rare cases (urinary and intracranial infections) missed by CTM. mNGS revealed mixed infections in 82.1% (46/56) of patients, substantially higher than the 5.4% detected by CTM. Notably, mNGS-guided therapy adjustments occurred in 74.1% of cases, compared with 22.2% for CTM (*p* < 0.001), correlating with clinical improvement in 90% (36/40) of adjusted regimens.

**Conclusion:**

Our data demonstrated that mNGS had a higher positive detection rate than CTM for detecting coinfections among PLWH, especially for *T. marneffei* and mixed infections. These results highlight the clinical value of mNGS as a complementary tool for pathogen identification in this vulnerable population.

## 1 Introduction

People living with HIV (PLWH) are highly susceptible to opportunistic infections by diverse pathogens due to compromised immunity (1–3). Despite advances in antiretroviral therapy, delayed diagnosis of opportunistic infections remains a critical obstacle to effective patient management. Conventional testing methods (CTM), including culture and smear microscopy, are limited by their restricted pathogen detection range, time-consuming procedures, and susceptibility to prior antibiotic use (4). Therefore, the development of novel diagnostic strategies is urgently needed in this vulnerable population.

The past decade has witnessed burgeoning interest in the application of metagenomic next-generation sequencing (mNGS) for microbial identification due to its unbiased approach, rapidity, and high sensitivity (5–8). By comparing sequencing data with specialized databases, mNGS theoretically enables clinicians to identify a wide range of microorganisms (9–13). In recent years, mNGS has been increasingly utilized in diagnosing infectious diseases and detecting resistance mutations of pathogens (11, 12, 14, 15). Invasive talaromycosis accounts for up to 15% of HIV-related hospital admissions in endemic regions such as Guangxi and Guangdong provinces, with the highest mortality rate among AIDS-associated complications (16). Recognized as a medium priority pathogen in the 2022 World Health Organization (WHO) Fungal Priority Pathogens List, *T. marneffei* poses diagnostic challenges due to nonspecific presentations (17). While several case reports have described mNGS detection of *T. marneffei* (18–22), the utility of mNGS for diagnosing *T. marneffei* infections among PLWH remains unclear.

In this study, we aimed to compare the pathogen diagnostic performance of mNGS with that of CTM, particularly in cases of *T. marneffei* and mixed infections. We further characterized the pathogen spectrum in PLWH in Southern China and assessed mNGS-guided therapeutic adjustments in hospitalized PLWH.

## 2 Materials and methods

### 2.1 Study design and subjects

This study enrolled 56 patients infected with HIV who were hospitalized at the Third Affiliated Hospital of Sun Yat-sen University from March 2022 to October 2024. The inclusion criteria for this study were as follows: (1) Patients diagnosed with HIV/AIDS by Western blot; (2) patients aged over 18 years; (3) patients whose primary diagnosis at discharge was an HIV-related infection; (4) patients who had completed conventional pathogen testing (smear, culture, or molecular methods) and mNGS testing; (5) patients with complete medical records, including laboratory testing data, documented treatment, and clinical outcome records. The exclusion criteria were: (1) patients with a combined diagnosis of a malignant tumor; (2) patients undergoing immunosuppressive therapy within the past 3 months; (3) patients with repeat hospital admissions within the study period.

In this study, detailed demographic information, clinical data, and pathogen detection results of the patients were obtained and documented from the electronic medical record system. Final diagnosis, serving as the reference standard, was determined by agreement of two independent infectious disease specialists based on a comprehensive review of all patient information. The Diagnosis of *T. marneffei* was established using a composite reference standard (CRS). This required the presence of criterion (i) and at least one additional criterion from (ii), (iii), or (iv) (23, 24): (i) Clinical manifestations such as fever, rash, weight loss, gastrointestinal abnormality, lymphadenopathy, hepatomegaly, splenomegaly, anemia, thrombocytopenia, and leukopenia. (ii) Positive cultures from specimens like blood, bronchoalveolar lavage fluid (BALF), cerebrospinal fluid (CSF), pus, or urine. (iii) Histopathological evidence of infection, with characteristic yeast cells identified in tissue biopsies via Periodic Acid-Schiff (PAS) or Wright’s staining. (iv) Detection of *T. marneffei* DNA by mNGS in blood, BALF, CNS, pus, or urine. For the primary analysis, we evaluated mNGS performance against this CRS. To address potential incorporation bias, we performed a pre-specified sensitivity analysis excluding criterion (iv), redefining the reference standard as clinical manifestations plus conventional testing methods only.

The criteria for adjusting medication regimens included the addition or discontinuation of antibacterial or antiviral agents, based on the results of pathogen examination and/or antimicrobial susceptibility testing. Therapeutic improvement was defined as the complete resolution of clinical symptoms (e.g., normalization of body temperature, cessation of productive cough) and laboratory markers, culminating in discharge with restored functional status. Treatment failure was defined as death or discharge against medical advice. The research protocol was approved by the ethics committee of the Third Affiliated Hospital of Sun Yat-sen University (No. II2025-035-01). Written informed consent was obtained from each participant.

### 2.2 Conventional pathogen testing

Peripheral blood samples, BALF, CSF, pus, and urine were collected from patients based on their symptoms and suspected sites of infection. Conventional diagnostic methods included bacterial culture, Gram staining, acid-fast staining, and reverse transcription polymerase chain reaction (RT-PCR). GeneXpert and T-SPOT assays were employed for the detection of *Mycobacterium tuberculosis*. Cryptococcus smear examination and Cryptococcus antigen testing were utilized for the identification of Cryptococcus species. PCR-based methods were applied for the detection of cytomegalovirus (CMV), Epstein–Barr virus (EBV), severe acute respiratory syndrome coronavirus 2 (SARS-CoV-2), influenza viruses, and hepatitis viruses.

### 2.3 mNGS platform

All mNGS procedures adhered to the standardized protocols established by the Department of Laboratory Medicine of the Chinese Medical Association for NGS. Genomic DNA isolation, enzymatic fragmentation, end repair, A-tailing, adapter ligation, and purification were performed using an automated NGS workstation. Library preparation and sequencing analysis were conducted on the Illumina Nextseq 550 DX platform, generating approximately 20 million raw paired-end reads per specimen. Raw data were cleaned by removing low-quality reads, adapter sequences, and fragments shorter than 50 bp and by filtering out human genome sequences (GRCh38.p13) using Bowtie2. Final pathogen identification was achieved by comparing filtered sequences with the proprietary Pathogen Genomic Database (DaAn Gene, Guangzhou, China). Criteria for positive mNGS results: (1) The species-specific strictly mapped read number (SMRN) per million ratios for *T. marneffei* was ≥1; (2) Other pathogens were identified as previously described (25).

### 2.4 Statistical analysis

Continuous variables were expressed as mean ± standard deviation (SD) for normally distributed data and as median (interquartile range, IQR) for non-normally distributed data. Comparisons of continuous variables between two groups were performed using the independent samples *t*-test for normally distributed data and the Mann–Whitney U test for non-normally distributed data. Categorical variables were analyzed using the chi-square test or Fisher’s exact test. Exact 95% confidence intervals for proportions were calculated using the Clopper-Pearson method implemented in R. All statistical analyses were conducted using SPSS version 23.0. Figures were generated using GraphPad Prism version 8.0 and R version 4.2.0.

## 3 Result

### 3.1 Clinical characteristics

**Figure 1:**
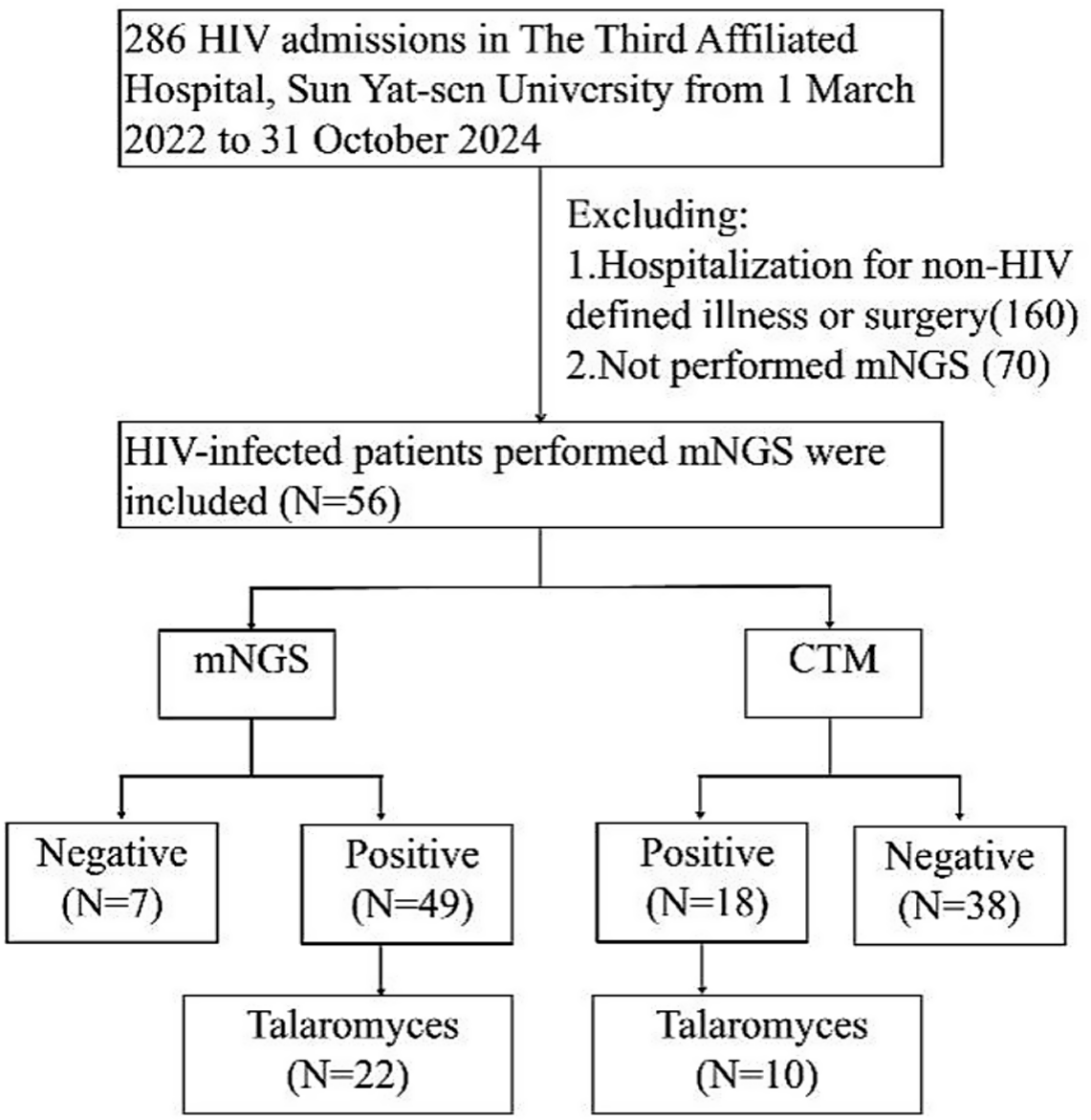
Study flowchart.

Between March 2022 and October 2024, a total of 286 PLWH were admitted to the Third Affiliated Hospital of Sun Yat-sen University. This study enrolled 56 patients with AIDS-related infections who underwent both CTM and mNGS testing (Figure 1). The characteristics of the participants are detailed in Table 1. The median age of the participants was 38.5 years, and the majority were male (91.0%). The primary symptoms observed were cough (75.0%), fever (69.6%), dyspnea (51.7%), lymphadenopathy (66.0%), weight loss (57.1%), rash (26.7%), hematological alterations (anemia, leukopenia, or thrombocytopenia, 26.7%), neurological manifestations (headache or altered consciousness, 25.0%), and diarrhea (21.4%). The participants exhibited elevated 1,3-*β*-D-glucan levels, with a median of 216.3 pg/mL (IQR: 78.5–563.0 pg/mL), and increased lactate dehydrogenase levels, with a median of 321 U/L (IQR: 230–461 U/L). A significant decrease was observed in both CD4^+^ T cell counts and CD4/CD8 T cell ratios among the participants, consistent with the typical features of HIV infection.

**Table 1:** Demography and symptomatology of participants.

Variable	Data
Age (years)	38.5 (30.3–54.8)
Male (%)	51 (91.0)
Symptoms	
Cough	75.0%
Fever	69.6%
Dyspnea	51.7%
Lymphadenopathy	66.0%
Weight loss	57.1%
Rash	26.7%
Hematological alterations	26.7%
Neurological symptoms	25.0%
Diarrhea	21.4%
Variable	
WBC (×10^9^/L)	5.71 (3.69–7.71)
Hemoglobin (g/L)	116 (96–130)
Neutrophil (×10^9^/L)	3.97 (2.43–6.03)
Platelet (×10^9^/L)	218 (135–293)
AST (U/L)	44 (26–73)
ALT (U/L)	33 (17–47)
eGFR	116.4 (96.3–129.2)
Lactate dehydrogenase (U/L)	321 (230–461)
1, 3*β*-D-glucan assays test (pg/mL)	216.3 (78.5–563.0)
CRP (mg/L)	33.9 (17.2–75.6)
Procalcitonin (ng/mL)	0.12 (0.05–0.51)
CD4 (cell/mm^3^)	32 (10–77)
CD8 (cell/mm^3^)	418 (208–664)
CD4/8 ratio	0.08 (0.04–0.19)
IgG (g/L)	19.9 (14.4–29.3)
IgA (g/L)	4.2 (2.8–6.8)
IgM (g/L)	1.5 (1.1–1.8)

Data are presented as median (inter quartile range) or the number of patients (percent).

### 3.2 Detection performances of CTM and mNGS

**Figure 2:**
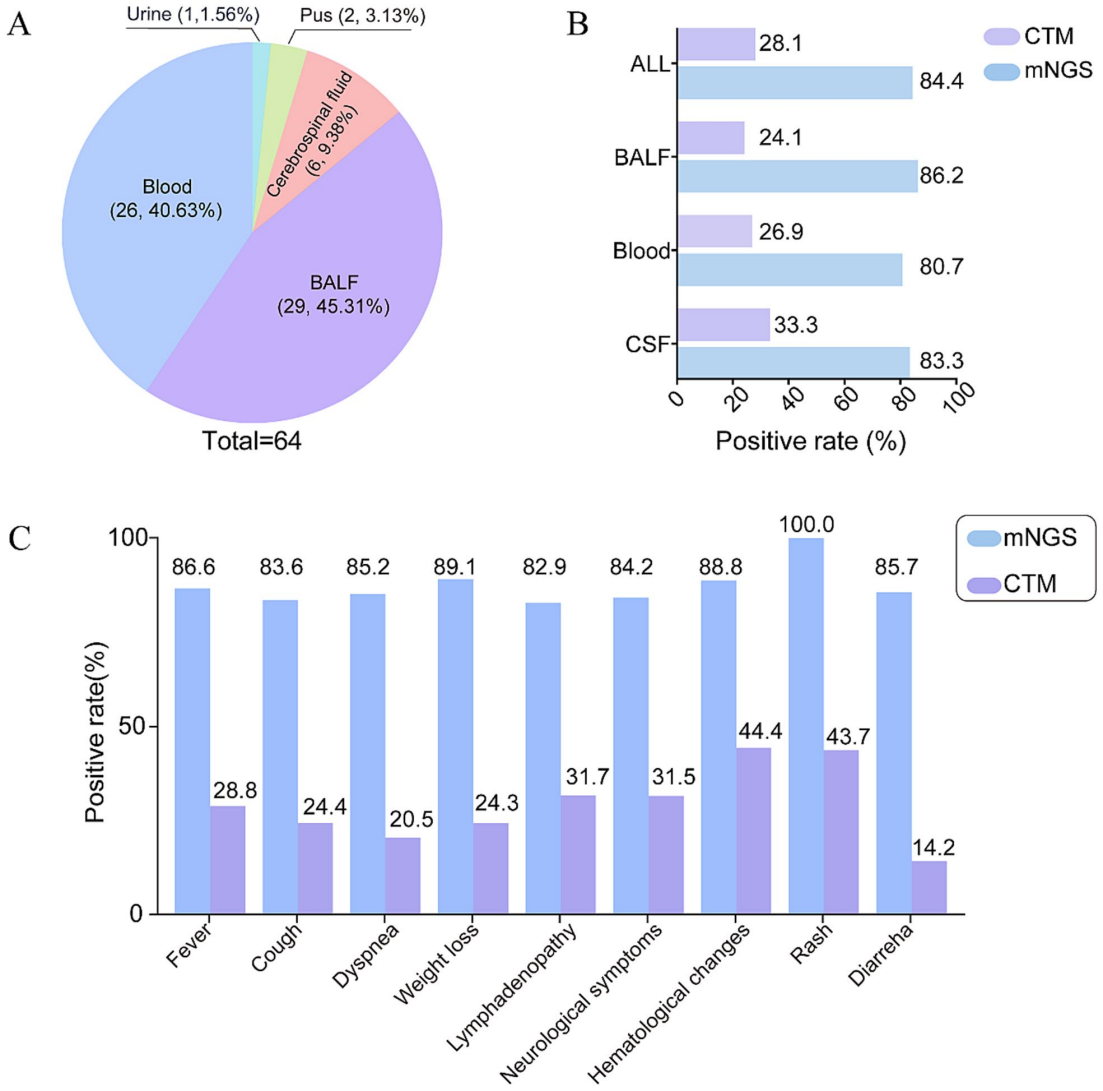
Detection performance of mNGS and CTM: **(A)** Types of specimens for mNGS testing; **(B)** positivity of mNGS and CTM across different specimen types; **(C)** positivity of mNGS and CTM in patients presenting with different symptoms. mNGS, metagenomic next-generation sequencing; CTM, conventional testing methods.

A total of 64 samples from 56 PLWH were analyzed by mNGS, including peripheral blood specimens (*n* = 26), BALF (*n* = 29), CSF (*n* = 6), pus (*n* = 2), and urine (*n* = 1) (Figure 2A). The overall pathogen detection rate was significantly higher with mNGS (84.4%, 54/64; 95% CI: 73.1–92.2%) than with CTM (28.1%, 18/64; 95% CI: 17.6–40.8%; *p* < 0.0001). This superior positivity rate of mNGS was consistently observed across different sample types (Figure 2B). We further compared the detection results of mNGS and CTM according to presenting symptoms (Figure 2C). Irrespective of the presenting symptoms, mNGS demonstrated higher positivity compared to CTM. Besides, in patients presenting with hematological symptoms and rash, the positivity of CTM was relatively higher.

**Figure 3:**
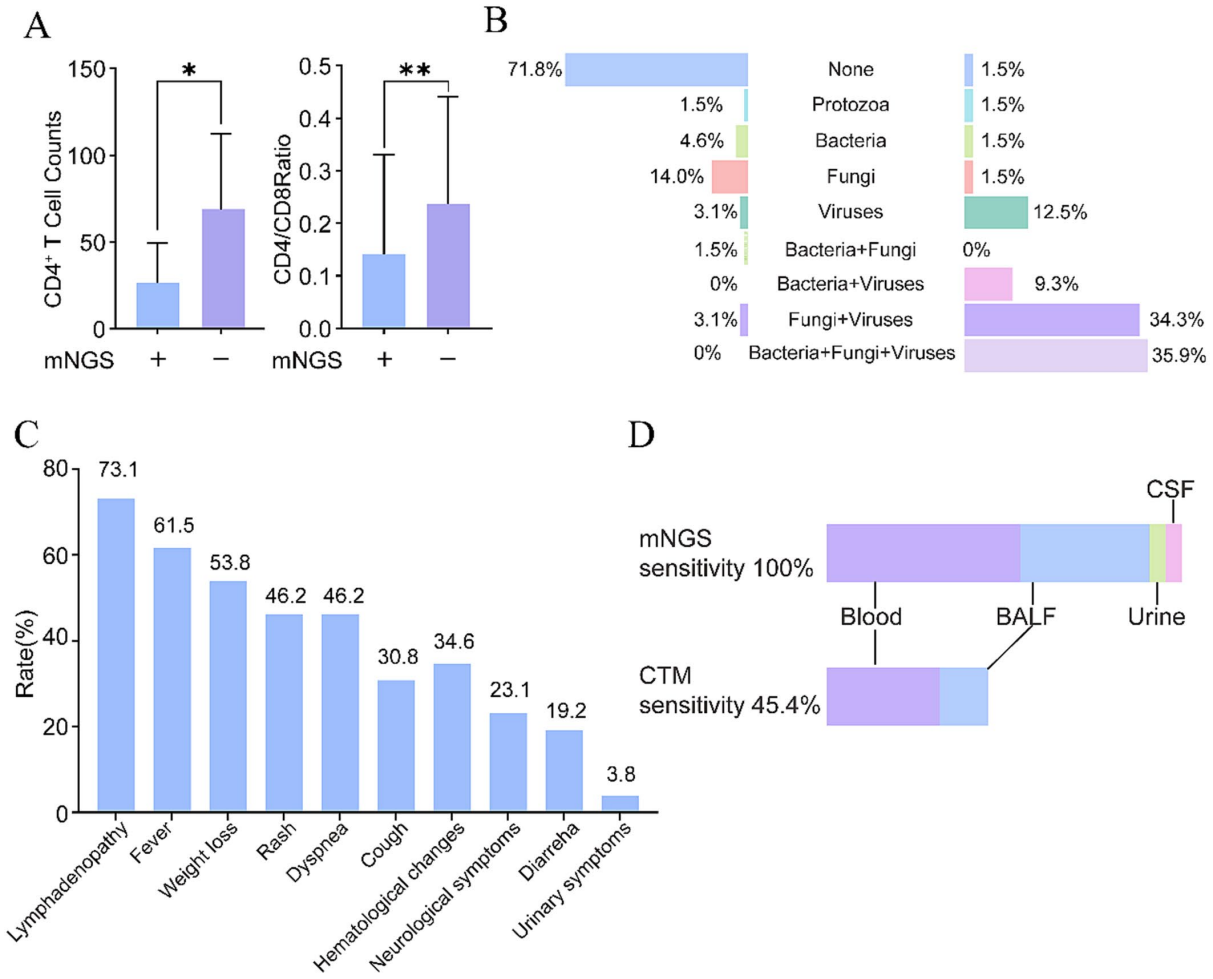
Detection performance of mNGS in mixed infections and *T. marneffei*: **(A)** Absolute CD4^+^ T cell counts and CD4^+^/CD8^+^ ratios in patients with positive and negative mNGS results; **(B)** proportions of mixed infections detected by mNGS and CTM; **(C)** clinical manifestations of *T. marneffei* infections in the study cohort; **(D)** detection performance of mNGS in *T. marneffei* infections. mNGS, metagenomic next-generation sequencing. *p < 0.05, **p < 0.001.

As illustrated in Figure 3A, patients with positive mNGS results exhibited significantly lower CD4^+^ T cell counts (*p* = 0.0147) and CD4/CD8 T cell ratios (*p* = 0.0091) compared to those with negative mNGS results. These findings highlight the potential influence of host immune status on the diagnostic efficiency of mNGS.

Due to their compromised immune systems, individuals with HIV are more susceptible to mixed infections. Among 64 samples from 56 PLWH, mNGS detected mixed infections in 51 samples from 46 individuals (82.1%) (Figure 3B), while conventional testing methods detected mixed infections in only 3 samples from 3 individuals (5.4%). The most common type of mixed infection was a combination of viral, bacterial, and fungal infections (*n* = 23), followed by coinfections of fungi and viruses (*n* = 22). These findings highlight the complexity of infections in individuals with HIV/AIDS and underscore the utility of mNGS in accurately identifying multiple pathogens.

### 3.3 *Talaromyces marneffei* infections detected by mNGS

*Talaromyces marneffei* was detected in 39.3% (22/56, 95% CI: 26.5–53.3%) of PLWH in our cohort (Supplementary Table S1). *Talaromyces marneffei* infection usually presents with atypical symptoms including lymphadenopathy, fever, weight loss, rash, dyspnea, and cough (Figure 3C). Importantly, mNGS demonstrated 100% positivity rate (22/22, 95% CI: 84.6–100.00%) across diverse specimen types: peripheral blood (*n* = 13), BALF (*n* = 8), CSF (*n* = 1), and urine (*n* = 1). In contrast, CTM showed markedly lower positivity (45.5%, 10/22, 95% CI: 24.4–67.8%, *p* < 0.0001), detecting the pathogen in 53.8% of blood cultures (7/13) and 37.5% of BALF cultures (3/8) (Figure 3D). To validate the diagnostic performance of mNGS independently, we conducted a sensitivity analysis using CTM positivity as the sole microbiological reference standard. Among 56 patients, 10 were confirmed to have *T. marneffei* infection. mNGS demonstrated a sensitivity of 100.0% (10/10) and a specificity of 73.9% (34/46; 95% CI: 58.9–85.7%). These results confirm that mNGS successfully identified all culture-positive cases. The 12 mNGS-positive/CTM-negative cases presented with compatible clinical syndromes (fever, weight loss, lymphadenopathy) and responded to antifungal therapy, suggesting true infections missed by conventional methods (Supplementary Table S1). Importantly, mNGS identified *T. marneffei* in two diagnostically challenging cases: (1) a patient with an 8-month history of urinary frequency and urgency, in whom conventional diagnostics failed to yield a definitive diagnosis, and (2) a patient presenting with 3 months of dizziness and ataxia, where the pathogen was detected by cerebrospinal fluid mNGS. Following the mNGS-guided diagnosis, both patients received targeted antifungal therapy with liposomal amphotericin B, leading to resolution of urinary and neurological symptoms within 2–3 weeks.

### 3.4 Pathogen profiles based on mNGS

In this study, a total of 50 pathogens were detected using mNGS among the participants, including bacteria (*n* = 21), fungi (*n* = 13), viruses (*n* = 15), and protozoa (*n* = 1). The most common pathogenic bacteria were *Haemophilus parainfluenzae* (*n* = 6), *Klebsiella pneumoniae* (*n* = 6), and *Streptococcus pneumoniae* (*n* = 6). The predominant fungi were *Pneumocystis jirovecii* (*n* = 26), followed by *T. marneffei* (*n* = 23) and *Candida tropicalis* (*n* = 6). The most frequently detected viruses were *Human betaherpesvirus 5* (CMV, *n* = 45) and *Human gammaherpesvirus 4* (EBV, *n* = 41), with *Torque teno virus* (*n* = 36) also commonly detected.

**Figure 4:**
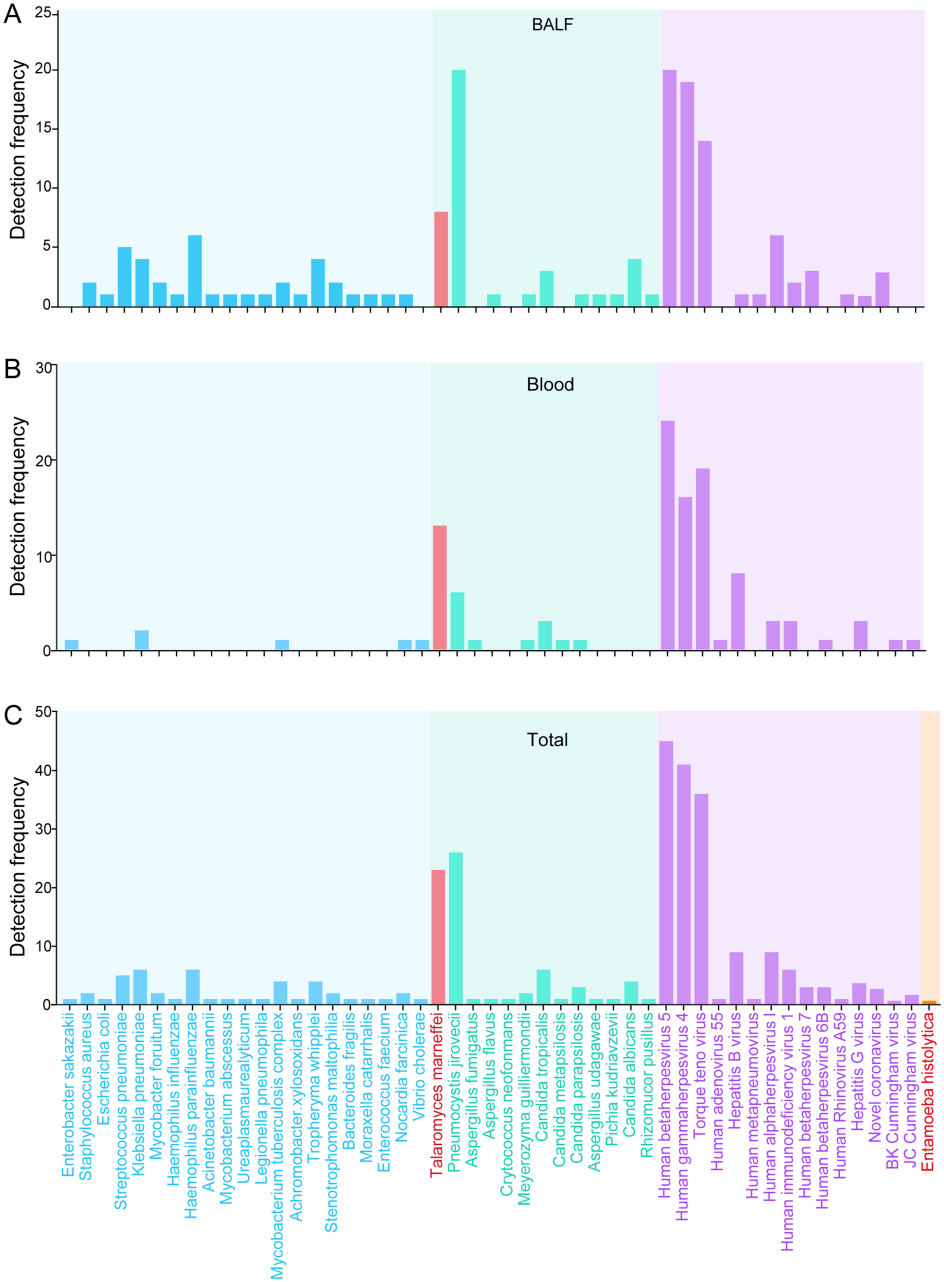
Pathogen spectrum identified by mNGS. **(A)** Pathogen spectrum detected by mNGS in bronchoalveolar lavage fluid (BALF). **(B)** Pathogen spectrum detected by mNGS in blood specimens. **(C)** Comprehensive pathogen spectrum detected by mNGS across all specimen types. mNGS, metagenomic next-generation sequencing.

As illustrated in Figure 4 and Supplementary Table S2, mNGS identified 40 pathogens in BALF samples and 23 pathogens in blood. Furthermore, mNGS was employed to detect Cryptococcus in two CSF samples. In addition, *T. marneffei*, CMV, and EBV were identified in three separate CSF samples using mNGS. Notably, the presence of *T. marneffei* was also confirmed in urine samples using mNGS. Meanwhile, *Entamoeba histolytica* was detected in pus aspirates from liver abscesses, and *Mycobacterium tuberculosis* was identified in pus aspirates from lymph node abscesses via mNGS.

Compared to mNGS, conventional detection methods identified a significantly lower number of pathogens. *Talaromyces marneffei* was the most frequently isolated pathogen. Other pathogens detected by traditional methods included a single instance of *Cryptococcus* identified through India ink staining, a case of tuberculosis confirmed by Xpert and T-SPOT assays, a positive result for CMV DNA, and a microscopic identification of *Entamoeba histolytica*. Overall, conventional pathogen detection methods exhibited limited ability to detect several fungi, including *Pneumocystis jirovecii*, *T. marneffei*, and Aspergillus. These results highlight that the mNGS platform demonstrated superior capability in revealing a wider range of pathogens compared to the culture-based platform in PLWH.

### 3.5 Influence of mNGS on diagnosis, antimicrobial therapy adjustment, and patient outcomes

**Figure 5:**
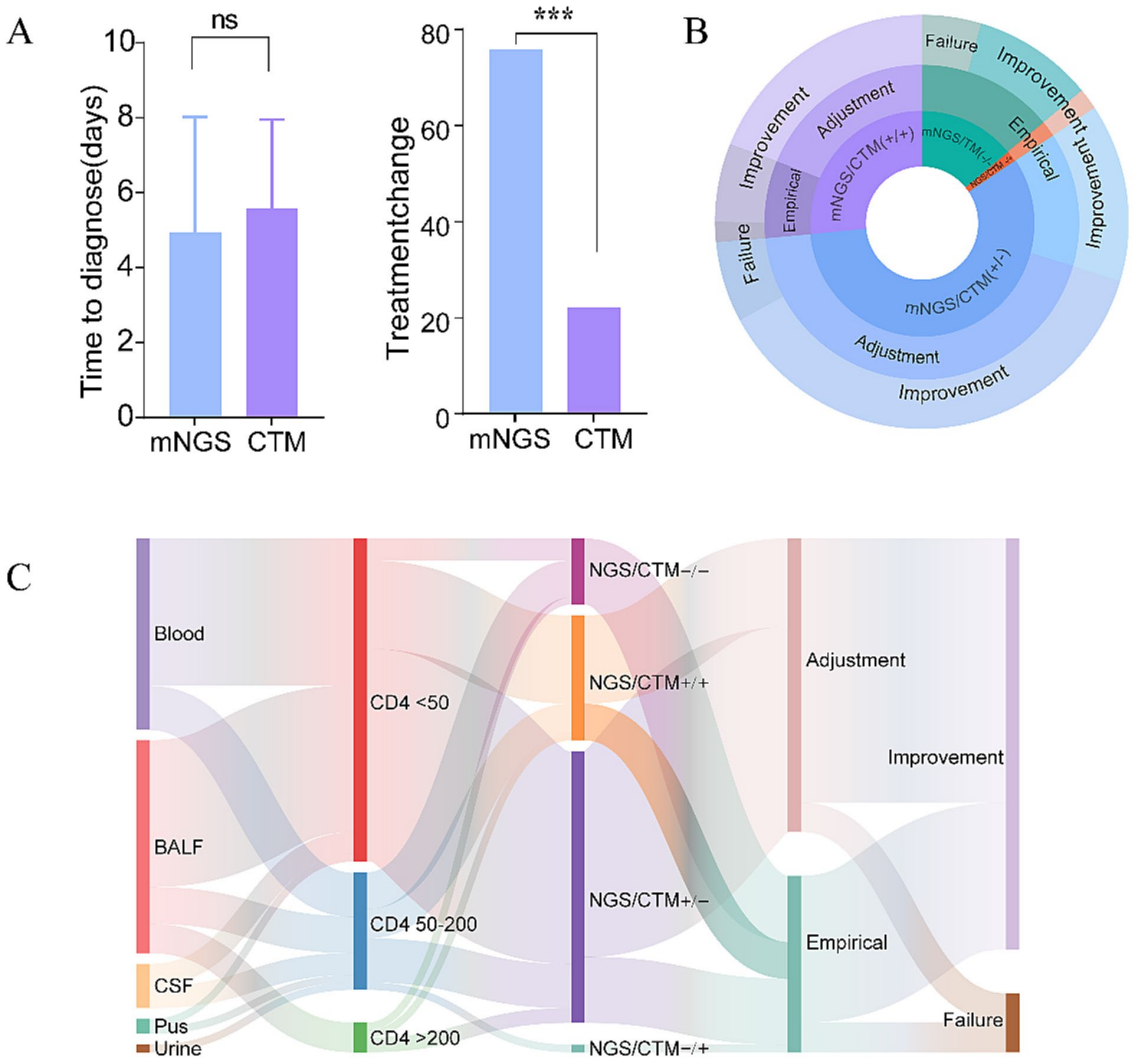
mNGS impact on diagnosis, antimicrobial therapy, and outcomes: **(A)** Time from hospital admission to pathogen diagnosis via mNGS or CTM; **(B)** proportion of treatment regimen adjustments based on mNGS and CTM results; **(C)** Sankey diagram illustrating pathogen detection and clinical decision pathways. mNGS, metagenomic next-generation sequencing; CTM, conventional testing methods. ***p < 0.0001.

The reporting time for mNGS was shorter than that of CTM (median: 1 day [IQR 1–2] vs. 4 days [IQR 3–6]; *p* < 0.0001). However, since specimen submission for mNGS testing was initiated later than CTM in clinical practice, no significant difference was observed in confirmation time (from hospital admission to definitive diagnosis) between the two methods (median: 4 days [IQR 2–8] vs. 5 days [IQR 4–7]; *p* = 0.315). Clinicians were significantly more likely to modify treatment regimens based on mNGS findings compared to CTM results (74.1% vs. 22.2%, *p* = 0.0009), indicating that mNGS information can effectively inform clinical decision-making (Figure 5A).

Empirical antimicrobial therapy was initiated at admission and adjusted based on mNGS or CTM results. Regimen modifications occurred in 70.6% (12/17) of mNGS+/CTM+ cases and 75.7% (28/37) of mNGS+/CTM− cases (Figure 5B). Empirical therapy was maintained throughout for mNGS−/CTM− (*n* = 9) and mNGS−/CTM+ (*n* = 1) cases. Treatment guided by mNGS results achieved 90% effectiveness (36/40), while empirical therapy alone resulted in 83.3% improvement (20/24).

Figure 5C shows the dynamic trajectories of 64 samples across five stages: sample, CD4^+^ T cell stratification, diagnostic concordance, treatment, and outcome. 93.2% (41/44, 95% CI: 81.3–98.6%) of severely immunocompromised individuals (CD4^+^ T cell <50 cells/mm^3^) were positive by mNGS or CTM, compared to 70% (14/20, 95% CI: 45.7–88.1%) in those with CD4^+^ T cell ≥50 cells/mm^3^ (*p* = 0.022). All mNGS−/CTM− patients received empirical therapy. Empirical antifungal escalation was similar in mNGS+/CTM− and mNGS+/CTM+ patients (12/17; 95% CI: 44.0–89.7%vs. 28/37; 95% CI: 58.8–88.2%, *p* = 0.467). One mNGS−/CTM+ patient, with a history of *tuberculosis*, received empirical anti-tuberculosis therapy before Xpert results. Most cases (36/40, 95% CI: 76.3–97.2%) achieved clinical remission after treatment adjustments. Treatment failures were mainly observed in severely immunodeficient patients with severe pneumonia. Empirical therapy failures were due to central nervous system infections or undetermined pathogens.

## 4 Discussion

In this study, we assessed the diagnostic utility of mNGS in PLWH. mNGS demonstrated high positivity rate and a broad pathogen detection spectrum compared to CTM, exhibiting good performance in identifying *T. marneffei* and mixed infections. This study revealed that the positivity rate of mNGS was significantly higher than that of CTM (84.4% vs. 28.1%, *p* < 0.0001) and comparable to previous studies (6, 26). Consistent with the literature (26, 27), our data demonstrated that host immunocompetence critically determines the diagnostic efficacy of mNGS, showing enhanced detection positivity in patients with lower CD4^+^ T cell counts.

Approximately 30% of *T. marneffei* remain undiagnosed or misdiagnosed attributed to the atypical clinical manifestations (28, 29). Central umbilicated rash is pathognomonic for *T. marneffei*, but is absent in up to 60% of patients (30). In our cohort, fewer than 50% presented with suggestive skin lesions, while nonspecific symptoms such as fever, cough, lymphadenopathy, and weight loss were more common, overlapping with other opportunistic infections. In our study, the detection rate of *T. marneffei* by mNGS was significantly higher than that by CTM, particularly in BALF samples (100% vs. 37.5%, *p* = 0.026), consistent with prior studies (31). While inclusion of mNGS in our reference standard may overestimate specificity, mNGS maintained perfect sensitivity (100%) and NPV (100%) in sensitivity analysis, confirming its value for excluding disease. The lower specificity (73.9%) and PPV (45.5%) likely reflect imperfect gold standards rather than false positives, supported by clinical findings and treatment response in mNGS-positive/CTM-negative cases. Notably, mNGS facilitated the diagnosis of two atypical *T. marneffei* cases involving the urinary tract and central nervous system, both of which had remained undiagnosed for several months following initial clinical assessment. Our findings emphasize the pressing need for the incorporation of mNGS into *T. marneffei* control programs within endemic regions.

mNGS also demonstrated a broader capacity for identifying potential pathogens. In this study, a therapy-refractory case initially misdiagnosed as a bacterial liver abscess was rapidly and accurately diagnosed as *Entamoeba histolytica* through mNGS analysis of pus samples. These findings demonstrate the enhanced detection capability of mNGS for fastidious pathogens and agents of neglected tropical diseases. Furthermore, mNGS offers a distinct advantage in diagnosing mixed infections due to its unbiased detection capabilities (26, 32). In our study, most patients (82.1%, *n* = 46/56) exhibited mixed infections, with the most common combinations being bacterial-fungal-viral and fungal-viral infections. This pattern may be explained by the high prevalence of respiratory infections in our cohort, with CMV and *Pneumocystis jirovecii* frequently detected in immunocompromised individuals with pulmonary infections (6, 20, 33, 34).

mNGS is limited in its ability to discriminate colonization from infection (35, 36). mNGS results require cautious interpretation to establish definitive diagnoses and avoid unnecessary treatment. In practice, clinical microbiologists categorized detected organisms by mNGS as pathogens of concern, suspected pathogens, or colonizing flora based on specimen type and clinical context (9); clinicians subsequently integrated these classifications with clinical manifestations, laboratory findings, and radiological evidence to guide treatment decisions. In our PLWH cohort, detection of *T. marneffei* or *Pneumocystis jirovecii* with compatible features led to antifungal therapy. By contrast, viral detections such as CMV were more difficult to interpret, illustrating that therapeutic decisions are inherently pathogen-specific.

This study has several limitations that should be acknowledged. First, the single-center design and relatively small sample size may introduce selection bias. Future prospective, multicenter studies with larger cohorts are warranted to directly compare the diagnostic accuracy of mNGS and CTM in PLWH. Second, the non-simultaneous application of mNGS and CTM precluded valid comparisons of their individual contributions to reduce the time to definitive diagnosis.

## 5 Conclusion

Our data demonstrated that mNGS had a higher positive detection rate than CTM for detecting coinfections among PLWH, especially for *T. marneffei* and mixed infections. These results highlight the clinical value of mNGS as a complementary tool for pathogen identification in this vulnerable population.

## Data Availability

The data presented in the study are deposited in the CNGBdb repository, accession number CNP0009096 (https://doi.org/10.26036/CNP0009096).
