# Supplementary material for: Metagenomic next-generation sequencing improves diagnosis of Talaromyces marneffei and mixed infections in HIV/AIDS patients: a retrospective study
Source: Front Med (Lausanne). 2026 Apr 7;13:1800314. doi: 10.3389/fmed.2026.1800314 (PMC13096071; doi:10.3389/fmed.2026.1800314)
Supplement: Supplementary file 1 [file Table_1.DOCX]

Supplementary Table 1

| **Case** | **Site of Infection** | **Symptom** | **CD4 T cell counts** | **Diagnose, time** | **Antimicrobial therapy** | **Hospitalization days, outcome** |
| --- | --- | --- | --- | --- | --- | --- |
| 26, Male | Blood, skin | Fever, cough, dyspnea, lymphadenopathy, weight loss, rash, thrombocytopenia, diarrhea | 7 | mNGS, 4 days Blood culture, 6 days | Voriconazole | 22 days, improved |
| 44, Male | Blood, skin, pulmonary | Fever, cough, lymphadenopathy, weight loss, rash, anaemia | 6 | BALF mNGS, 10 days Blood culture, 4 days | Amphotericin B, voriconazole | 14 days, improved |
| 29, Male | Pulmonary | Fever, cough, dyspnea, lymphadenopathy, weight loss | 25 | BALF mNGS, 8 days | Voriconazole | 16 days, improved |
| 33, Male | Pulmonary | Cough, dyspnea, lymphadenopathy | 33 | BALF mNGS, 8 days | Voriconazole | 10 days, improved |
| 29, Male | Blood | Cough, fever, weight loss, rash, lymphadenopathy, weight loss | 123 | mNGS, 4 days Blood culture, 9 days | Voriconazole, amphotericin B | 17 days, improved |
| 39, Male | Pulmonary, skin | Cough, fever, dyspnea, weight loss, rash, lymphadenopathy, weight loss, anaemia | 9 | BALF mNGS, 3 days | Voriconazole | 20 days, improved |
| 24, Male | Pulmonary | Cough, fever, dyspnea, weight loss, lymphadenopathy, diarrhea | 38 | BALF mNGS, 4 days | Voriconazole | 16 days, improved |
| 33, Male | Blood, pulmonary | Cough, fever, dyspnea, weight loss, rash, lymphadenopathy, anaemia | 7 | BALF mNGS, 9 days Blood culture, 5 days | Voriconazole | 12 days, improved |
| 58, Male | Urinary | Urinary frequency and urgency | 190 | Urine mNGS, 12 days | Voriconazole, amphotericin B | 30 days, improved |
| 25, Male | Pulmonary | Cough, fever, dyspnea, rash, lymphadenopathy, headache | 16 | Urine mNGS, 12 days | Amphotericin B | 14 days, improved |
| 57, Male | Blood | Dyspnea, rash, weight loss, lymphadenopathy | 7 | mNGS, 2 days Blood culture, 5 days | Amphotericin B | 4 days, improved |
| 63, Female | Blood | Fever, rash, lymphadenopathy, anaemia, thrombocytopenia | 6 | mNGS, 2 days Blood culture, 4 days | Voriconazole, amphotericin B | 20 days, improved |
| 24, male | Blood | Cough, fever, dyspnea, weight loss | 13 | mNGS, 3 days | Amphotericin B, amphotericin B Cholesteryl Sulfate Complex | 11 days, improved |
| 32, female | Blood | Abdominal pain, diarrhea, lymphadenopathy, anaemia | 54 | mNGS, 3 days Blood culture, 5 days | Amphotericin B, amphotericin B Cholesteryl Sulfate Complex | 23 days, improved |
| 53, male | Blood | Cough, fever, dyspnea, lymphadenopathy, weight loss, headache | 7 | mNGS, 2 days  Blood culture, 4 days | Amphotericin B | 19 days, improved |
| 25, male | Blood, Skin | Rash, fever, cough, dyspnea, lymphadenopathy, weight loss | 48 | mNGS, 2 days | Amphotericin B | 20 days, improved |
| 60, male | Blood, pulmonary | Fever, cough, abdominal pain, diarrhea, lymphadenopathy, weight loss, headache, thrombocytopenia | 39 | BALF mNGS, 3 days | Amphotericin B | 20 days, improved |
| 30, male | Blood | Fever, cough, dyspnea, diarrhea, lymphadenopathy, weight loss, headache | 23 | mNGS, 3 days | Amphotericin B | 23 days, improved |
| 37, male | Blood, pulmonary | Unsteady gait, headache, vomiting, lymphadenopathy, | 158 | CSF mNGS, 2 days | Amphotericin B, Amphotericin B Cholesteryl Sulfate Complex | 42 days, improved |
| 34, male | Blood, skin | Rash, pharyngalgia | 61 | mNGS, 2 days | Amphotericin B | 16 days, improved |
| 46, male | Blood, skin | Fever, cough, lymphadenopathy, weight loss, rash, neutropenia | 2 | mNGS, 2 days Blood culture, 3 days | Amphotericin B, Amphotericin B Cholesteryl Sulfate Complex | 13 days, improved |
| 46, male | Blood | Fever, lymphadenopathy, dizziness, headache, and blurred vision, anemia | 9 | mNGS, 3 days | Amphotericin B | 17 days, improved |
